# Abridging the CMOS Technology II

**DOI:** 10.3390/nano14110897

**Published:** 2024-05-21

**Authors:** Hei Wong

**Affiliations:** Department of Electrical Engineering, City University of Hong Kong, Hong Kong, China; heiwong@ieee.org

As silicon CMOS devices approach their physical and technological miniaturization limits, Moore’s Law is expected to persist for several more cycles, thanks to innovative, more compact layout structures. These advancements include the development of nanosheet, forksheet, and CFET device structures alongside the reconfiguration of power rails in a vertical direction and on the backside of the chip, as well as 3D stacking techniques that extend from individual devices to whole systems [1,2,3,4,5,6]. Meanwhile, the emergence of groundbreaking 2D semiconductor materials constitutes the most promising option for the continued evolution of integrated electronics. Nevertheless, achieving the integration of 2D material ICs that would rival current state-of-the-art CMOS technology remains a distant goal. It is anticipated that CMOS technology will continue to be the key integration technology for the foreseeable future, as new materials and devices are not imminently expected to replace CMOS technology. Given this background, this collection of articles, titled “Abridging CMOS Technology II”, follows the success of the first Special Issue, “Abridging the CMOS Technology” [7], and provides a platform for multidisciplinary discussions on the latest progress in nano CMOS technology, as well as innovative 2D materials and nanotechnology poised to abridge future CMOS technology advancements. In total, this archive consists of three comprehensive review papers and eight regular papers.

Regardless of nano CMOS technology or electronic devices based on 2D materials, contact scaling poses significant challenges due to several undesirable factors such as surface roughness, reduced contact size, and lower doping concentration, which become more pronounced at nanometer scales. These issues could therefore hinder the further advancement of CMOS device downsizing. At the same time, metal/2D material contact has long been a crucial issue concerning electronic device applications. In their review paper entitled “Contacts at the Nanoscale and for Nanomaterials” (contribution 1), Wong, Zhang, and Liu address contact issues in both of these domains. They address the implications of contact size reduction from perspectives of physics and technology, further highlighting the challenge of achieving transparent ohmic contacts with 2D materials, which arises from the limitations of doping technology, metal selection for band alignment, Fermi-level pinning, and the van der Waals gap. Their paper concludes by outlining several promising strategies to overcome these barriers, including hybridization and edge contacts, junction doping, alterations in phase and bandgap, and the introduction of buffer layers—all aimed at establishing good ohmic contacts.

Three contributions to this Special Issue subsequently concern nano CMOS device structures themselves. Liu and colleagues investigated the reliability concerns associated with FinFETs (contribution 2), discovering that the degradation of FinFET device characteristics due to hot carriers is correlated with flicker noise levels. Additionally, they developed a coupling model for characterizing trap time constants, which could be instrumental in profiling trap energy distribution. The reduction in the size of the nanowire/nanosheet leads to a decrease in the silicon channel’s cross-section to dimensions nearing the interface transition layer width, resulting in notable device performance degradation due to predominant surface roughness scattering effects on charge transport. Elmourabit and associates additionally delve into the carrier distribution within the 2D quantized metal/insulator transition region (contribution 3). Their conductance measurements reveal two distinct behaviors, metallic and insulating, which are governed by critical carrier density. The presence of a high-defect-density surface introduces a unique charge transport mechanism that deviates from the traditional drift model. Thus, Wong and Kakushima introduced an innovative device structure that utilizes lateral Poole–Frenkel charge transport through surface defects (contribution 4). The PF-MOS transistor described offers the benefits of reduced power dissipation and the potential to overcome technological limitations related to channel mobility degradation and gate oxide scaling, coming to be considered the ultimate MOS structure.

There is a consensus that the next “more Moore” should be 3D stacking and the heterogeneous integration of multiple chiplets. Specifically, architectures such as “near-memory computing” are anticipated to enhance data processing speeds while reducing power consumption [3]. Notably, vertical stacking has advanced considerably in memory stacking due to its relatively minor heat dissipation challenges. In this context, Spassov and Paskaleva conducted an extensive review of flash memory technology utilizing HfO_2_/Al_2_O_3_ nanolaminate stacks (contribution 5). Their findings suggest that these nanolaminated stacks can be custom engineered in order to create specific trap levels and to modulate their energy and spatial distributions by varying the aluminum content in HfO_2_. These authors provide a detailed analysis of how the composition of the charge-trapping layer, annealing processes, and the thickness of the tunneling oxide can affect the memory window, retention, and endurance of flash memory. They also shed light on potential future research directions and innovative developments that may arise from HfO_2_/Al_2_O_3_-based charge-trapping stacks. 

Zinc oxide (ZnO) is recognized as a wide bandgap semiconductor, boasting several distinctive characteristics not found in CMOS technology, and it has been extensively utilized in a diverse array of sensory devices. Moreover, ZnO shows great potential as a material for flexible thin-film transistors (TFTs). Thus, this anthology includes a contribution by Wang et al., who reported on a novel ZnO:H/ZnO dual-layer structure aimed at improving a device’s performance (contribution 6). Their findings suggest that introducing hydrogen into the structure can mitigate oxygen-related defects, thereby diminishing carrier scattering and concurrently increasing carrier density.

As nanodevices are anticipated to exhibit increased vulnerability to electrostatic discharge (ESD) strikes, developing an effective ESD protection strategy that fits within a reduced design space and meets stringent requirements remains a significant challenge. A review by Li et al. (contribution 7) addresses these concerns, presenting a range of graphene-based ESD solutions that exemplify the integration of nanomaterials with CMOS technology. Furthermore, this Special Issue contains three contributions that report on device innovation with emerging 2D semiconductor materials. Abidi and co-workers developed a terahertz signal detection circuit using asymmetric dual-grating-gate bilayer graphene FETs (contribution 8), achieving nearly an order of magnitude increase in the photocurrent, which was attributed to the modulation of hole and electron concentrations via top and back gate biasing. Moreover, Xia et al. present a report of a brain implant electrode innovatively designed using single-walled carbon nanotubes (SWCNTs) (contribution 9). These SWCNTs are advantageous due to their minimal footprint, reduced impedance, and enhanced signal-to-noise ratio, especially when benchmarked against traditional probes. Wong et al. additionally present simulation work on a carbon nanotube (CNT)-encapsulated carbyne structure (contribution 10), with their findings suggesting that the stability of the carbyne nanotube is influenced by several factors, including the inherent characteristics of the CNT, its porosity, the external pressure applied, the ambient temperature, and the length of the carbyne chain. These dependencies suggest the possibility of carbyne length optimization for CNT transistors. Collectively, these reports reveal some of the various potential applications of 2D materials for heterogeneous CMOS integration.

CMOS technology has been the most powerful engine propelling our society into the digital and intelligent era. While the miniaturization of CMOS devices is approaching its physical limits, CMOS will remain the predominant integration technology for the foreseeable future. The next leap in technological advancement, therefore, is expected to stem from integrating innovative 2D materials into existing CMOS processes, thereby boosting circuit performance and introducing new functionalities. This Special Issue series thus aims to serve as a platform for experts from various disciplines to share their latest research results in this area. Both Special Issues, featuring six review papers and thirteen original contributions combined, collectively address the most significant topics within this field.
